# Assessing quality of life with SarQol is useful in screening for sarcopenia and sarcopenic obesity in older women

**DOI:** 10.1007/s40520-023-02488-7

**Published:** 2023-07-13

**Authors:** Rosa Fonfría-Vivas, Pilar Pérez-Ros, Joaquín Barrachina-Igual, Ana Pablos-Monzó, Francisco M. Martínez-Arnau

**Affiliations:** 1 Department of Nursing, Universitat de València, Av. Menendez Pelayo 19, 46010 Valencia, Spain; 2 Frailty and Cognitive Impairment Research Group (FROG), University of Valencia, Menéndez I Pelayo, 19, 46010 Valencia, Spain; 3 Faculty of Physical Activity and Sport Sciences, Universidad Católica de Valencia San Vicente Mártir, Ramiro de Maetzu 14, 46900 Torrent, Valencia Spain; 4 Department of Physiotherapy, Universitat de Valencia, Gascó Oliag 5, 46010 Valencia, Spain

**Keywords:** Sarcopenia, Women, Sarcopenic obesity, Quality of life, Prevalence, SarQoL

## Abstract

**Background:**

Health-related quality of life (HRQoL) may be impaired in the presence of sarcopenia. Since a specific quality of life questionnaire became available for sarcopenia (SarQol), cutoffs to screen for this condition have been proposed, prompting the need to assess them in different populations. Due to the lack of consensus on diagnostic criteria, the tool has not yet been analyzed in screening for sarcopenic obesity.

**Aim:**

Our aim is to measure the SarQoL’s metric properties and establish a cutoff in QoL assessments that could be used along the diagnostic pathway for sarcopenia and sarcopenic obesity in community-dwelling older women.

**Methods:**

This cross-sectional study assessed women aged ≥ 70 years using the SarQol, sarcopenia criteria (EWGSOP2) and sarcopenic obesity criteria (ESPEN/EASO). Cutoffs for the SarQol were defined with a receiver-operating characteristics (ROC) curve, and sensitivity and specificity were analyzed.

**Results:**

Of the 95 included women (mean age 76.0 years, standard deviation [SD] 5.7), 7.3% (*n = *7) were classified as having sarcopenic obesity, 22.1% (*n = *21) as having sarcopenia, and 70.5% (*n = *67) as not having sarcopenia. The total SarQol score was higher in women without sarcopenia (66.5 SD 16.2) versus those with sarcopenia (56.6 SD 15.6) and sarcopenic obesity (45.1 SD 7.9). A cutoff of ≤ 60 points is proposed for sarcopenia screening (area under the ROC curve [AUC] 0.67; 95% confidence interval [CI] 0.53–0.80; sensitivity 61.9%; specificity 62%), and ≤ 50 points for sarcopenic obesity (AUC 0.85; 95% CI 0.74–0.95; sensitivity 71.4%; specificity 76.9%).

**Conclusions:**

Quality of life is compromised in women with sarcopenia and especially in those with sarcopenic obesity. The SarQol could be useful in screening for these conditions, providing insight into health-related quality of life in older people with sarcopenia.

## Introduction

Sarcopenia, defined as a decrease in muscle mass and strength, has an important impact on physical performance in older people [1]. Early detection is critical for proper management, making it essential to have criteria that can be routinely used in clinical practice. In this regard, the European Working Group on Sarcopenia in Older People (EWGSOP) proposed an updated diagnostic pathway [2], known as EWGSOP2 criteria. According to this tool, current prevalence of sarcopenia is around 11% (range 3.2% and 26.3%) in older adults [3], which is concerning given its negative impact on quality of life [4], the associated increase in complications, and the increased pressure on health systems [5].

At the same time, the prevalence of obesity is about 40.1% in older women and 32.5% in older men [6], and it is not uncommon for it to coexist with sarcopenia. This concurrence constitutes a clinical and pathophysiological entity of great complexity, exceeding the sum of the negative effects of both entities. This condition, known as sarcopenic obesity (SO), triggers pathophysiological mechanisms including insulin resistance, systemic inflammation, and oxidative stress, among others; sarcopenia and obesity mutually feedback on each other, multiplying their detrimental effects on strength and muscle mass [7] and increasing the risk of comorbidities (type 2 diabetes, osteoporosis, cognitive impairment, etc.) and all-cause mortality [8]. All this, although of great concern to the EWGSOP, did not immediately lead to the establishment of a specific diagnostic pathway for SO, thus favoring the proliferation of different diagnostic approaches in research [9].

Very recently, thanks to the joint work of the European Society for Clinical Nutrition and Metabolism (ESPEN) and the European Association for the Study of Obesity (EASO), the first screening and diagnostic criteria with specific cutoff values for SO have been published [10]. This instrument can facilitate early diagnosis and help establish the clinical relevance of SO along with its functional implications and impact on patients’ quality of life.

Regarding this last essential aspect, people with SO seem to have a poorer quality of life than those with obesity alone [11, 12]. On the other hand, some research comparing quality of life in people with SO versus sarcopenia alone shows no significant differences [13] or even better quality of life in people with SO [14].

However, these previous studies used different diagnostic criteria for SO than those recently established by ESPEN/EASO [10], so it is pertinent to study quality of life in older adults using the new diagnostic criteria for SO as well as the EWGSOP2 criteria [2]. Moreover, as recommended by Tsekoura et al. [4], such assessments should be undertaken using a specific tool, such as the Sarcopenia and Quality of Life (SarQol) instrument [15], whose different cross-cultural versions have shown very good structural and psychometric properties in people with sarcopenia [16].

The application of the sarcopenia assessment algorithm, based on the EWGSOP2 criteria, and the sarcopenic obesity algorithm, based on the EASO/ESPEN criteria, is relatively time-consuming and resource-intensive, underscoring the need for more abbreviated screening instruments such as the Sarc-F or the Mini Sarcopenia Risk Assessment (MSRA). Health-related quality of life could also be used for screening, and since a specific QoL questionnaire (SarQol) became available, several cutoff points have been proposed, from ≤ 52.4 points [17] to ≤ 60 points [18]. These thresholds could be useful for screening older people with sarcopenia, providing an acceptable screening accuracy in relation to Sarc-F. That said, confirming the cutoffs with other population cohorts is still necessary [17].

A recent validation study of the SarQol in Spanish showed good metric properties [19], and a cutoff of ≤ 60 points has been proposed in the Spanish population [18]. Given the need to confirm these cutoffs in women with sarcopenic obesity, the primary aim of this study is to compare quality of life in older women without sarcopenia, with sarcopenia, and with sarcopenic obesity, and to measure the SarQoL’s metric properties in order to establish a cutoff in QoL assessments that could be used in the diagnostic pathways for these conditions in community-dwelling older women.

## Methods

This cross-sectional study included community-dwelling women aged 70 years and older who were able to stand independently during the time of the body composition assessment using bioelectrical impedance analysis (BIA). Exclusion criteria were as follows: use of a pacemaker or implanted defibrillator; diagnosis of advanced dementia (Global Deterioration Scale score of 7) or terminal illness (life expectancy < 6 months); presence of edema or hydration disorders that could affect BIA results; degenerative muscle diseases; and the presence of any other condition that could entail a risk to the participant.

Recruitment took place from April to December 2021 in primary healthcare centers and social centers for older people. Once recruited, the women were scheduled within 30 days for further clinical assessment at the University of Valencia Faculty of Physiotherapy. In total, 104 women were assessed, of whom 101 met the inclusion criteria and were contacted for eligibility. Six declined to participate, leaving a final sample of 95 women.

### Sample size

According to the EWGSOP2 criteria, the prevalence of sarcopenia in community-dwelling older people in one Mediterranean area was 3.2% [20]. Assuming a margin of error of 5% and a confidence level of 95%, the required sample size was 53 people (G-Power, Dusseldorf, Germany).

### Measurements

All assessments were performed by the same team, consisting of two nurses, a physiotherapist, and two physical activity professionals. Variables collected were as follows:

- Sarcopenia prediction outcome, using the SARC-F questionnaire.
- Anthropometric variables and body composition. Height was measured with a stadiometer (SECA 200 scale with built-in stadiometer, Hamburg, Germany), and waist circunference with a single metric tape. For the assessment of body composition, we used the BC-418-MA BIA device (Tanita 2016, America). Prior to the assessment, we verified that participants had not performed any prior physical exercise; that they had fasted for 2–3 h, abstaining from alcohol or large amounts of water, and that their bladder was empty; and that every metal item had been removed. To quantify muscle mass, the Skeletal Muscle Mass Index (SMMI) and the skeletal muscle mass adjusted for weight (SMM/W) were calculated as muscle mass/height^2^ (kg/m^2^) and as muscle mass/weight (kg/kg), respectively. The total percentage of fat mass was also recorded, as was body mass index (BMI).
- Muscle strength. Handgrip assessment of the dominant hand was performed using the Jamar Hydraulic Hand Dynamometer 5030J1 (Loughborough, UK), and the highest value out of three assessments was recorded. The 5 times-sit-to-stand (5XSTS) test was also performed, recording the seconds needed to transfer from a seated to standing position and back again five times as quickly as possible [21].
- Physical performance outcomes. Walking speed was evaluated by having participants walk at their usual pace for 4 m along a corridor, using a technical aid if needed. This test was performed twice, and the fastest time was recorded [1]. Participants also underwent the Short Physical Performance Battery (SPPB), which consists of three tests (balance, walking speed, and sit-to-stand), following the instructions detailed by Guralnik et al. [22].
- Health-related quality of life (HRQoL). The generic EuroQol, a scale with 5 dimensions and 3 levels (EQ-5D-3L) [23], was used, together with a specific scale for sarcopenia, SarQol [15]. The EQ-5D-3L assesses health status according to a descriptive system (questionnaire) and a visual analog scale (VAS). The dimensions evaluated are mobility, self-care, usual activities, pain and discomfort, and anxiety and depression. Each dimension has three levels of severity: no problems, moderate problems, and serious problems, thus providing a final set of 243 health states. The range of the scale goes from negative values, which indicate a quality of life worse than being dead, to 1, which indicates the highest possible quality of life [24]. The 20-cm VAS, with marked mm, ranges from 0 (worst imaginable state of health) to 100 (best imaginable state of health). Along the vertical line, the individual must mark the point that best reflects their overall self-assessed health status.

The SarQol is a self-administered questionnaire, with 22 questions covering 55 aspects of quality of life, organized around seven domains: physical and mental health, mobility, body composition, functionality, activities of daily living, leisure activities, and fears. It is scored on a scale from 0 to 100, with higher scores indicating better quality of life. This instrument has been validated in more than 35 languages. A Spanish psychometric validation of SarQoL [19] shows similar psychometric properties to those of the original version of the instrument [15].

- Function. This parameter was assessed using the Barthel scale, making it possible to determine a person’s functionality when performing 10 basic activities of daily living, for example, eating, toileting, going to the toilet, moving around, or dressing. Each activity is evaluated with a score of 10, 5 or 0, depending on the degree of help needed. The score ranges from 0 to 100, with 100 points indicating maximum autonomy [25].
- Nutritional status. This outcome was assessed using the Mini Nutritional Assessment (MNA), a hetero-administered scale for assessing nutritional status. The full scale has a range of scores from 0 to 30, with less than 17 points indicating malnutrition [26].
- Physical activity level. This outcome was assessed using the International Physical Activity Questionnaire (IPAQ) [27]. The four signaling questions elicit responses regarding the duration and frequency of vigorous, moderate, and light intensity activities, plus the duration of daily sitting time in hours. Metabolic equivalent of task (MET) values were calculated.

**Fig. 1:**
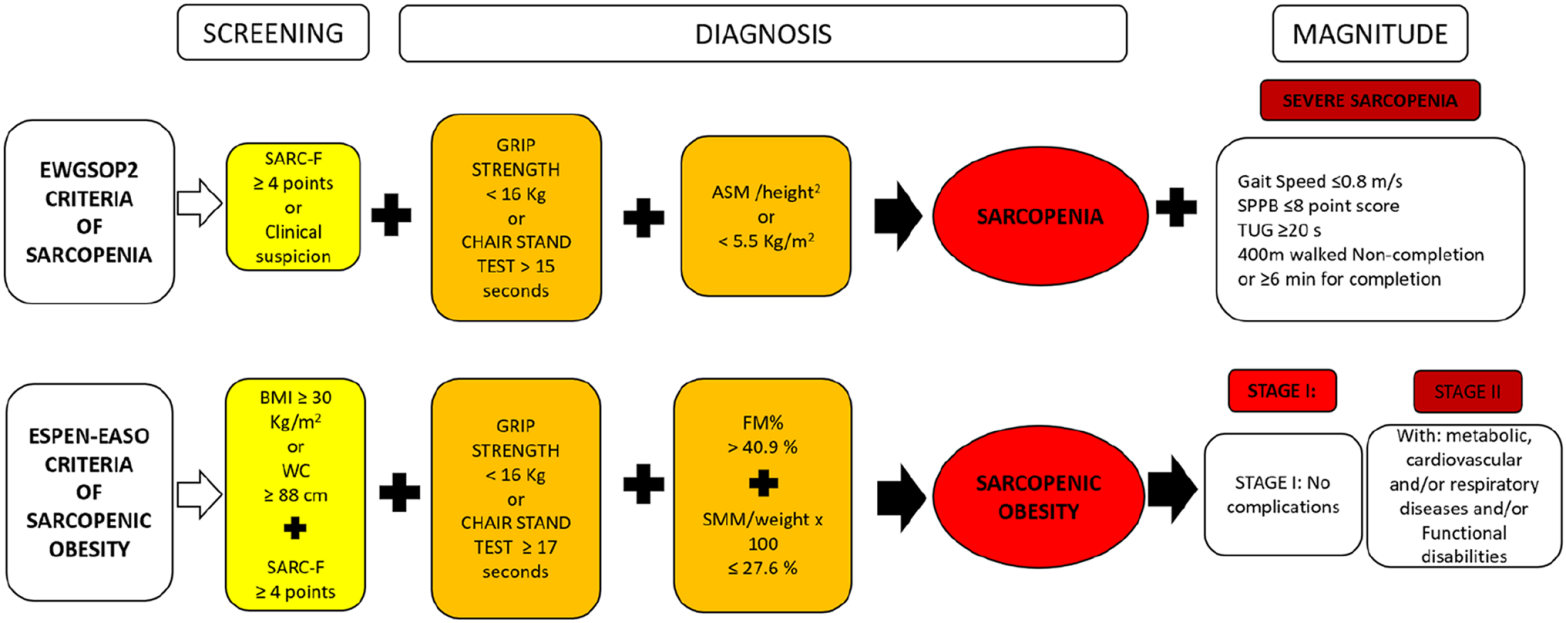
Sarcopenia and sarcopenic obesity screening and diagnostic criteria. *ASM* appendicular skeletal muscle mass; *BMI* body mass index; *ESPEN-EASO* European Society for Clinical Nutrition and Metabolism-European Association for the Study of Obesity; *EWGSOP2* European Working Group on Sarcopenia in Older People; *FM* fat mass; SMM; skeletal muscle mass; *SPPB* Short Physical Performance Battery; *TUG* timed up and go test; *WC* waist circumference

We screened for sarcopenia using the EWGSOP2 algorithm [2]. A positive screening was defined as Sarc-F score of ≥ 4 points or clinical suspicion. Probable sarcopenia was determined as grip strength < 16 kg or chair stand > 15 s, and this diagnosis was confirmed when low quantity muscle was also detected (SMMI < 5.5 kg/m^2^). Sarcopenia was considered severe in the presence of low physical performance (gait speed < 0.8 m/s; SPPB ≤ 8 points) (Fig. 1).

To assess sarcopenic obesity, participants were classified according to the EASO-ESPEN algorithm. A positive screening test was defined as follows: Sarc-F ≥ 4 points, clinical symptoms or suspicion, and BMI ≥ 30 kg/m^2^ or waist circumference ≥ 88 cm. Diagnostic confirmation was performed in two consecutive steps: the first criterion was altered skeletal muscle functional parameters (grip strength < 16 kg or chair stand > 15 s), and the second was altered body composition (total skeletal muscle mass adjusted by weight [SMM/W] or increased percentage of fat mass [> 40.9%]) [28]. From there, staging was undertaken: participants with no complications were classified as stage I, while those with at least one complication attributable to SO, such as metabolic diseases, functional disabilities, or cardiovascular and respiratory diseases, were considered to have stage II sarcopenic obesity **(**Fig. 1**).**

### Metric properties of SarQol

Feasibility was defined as less than 10% of missing data and over 95% of computable data [29]. Acceptability was defined as similar mean, median, and standard deviation values across items (15% as the maximum divergence) [30], in addition to values of asymmetry and kurtosis between − 1 and 1 [31]. Finally, the floor and ceiling effect (percentage of scores in the lower and upper extremes) had to be under 15% [32]. Reliability was analyzed according to the internal consistency and considered acceptable at Cronbach’s alpha ≥ 0.7 [32] Convergent validity is part of construct validity and examines whether an instrument correlates with other instruments to the degree that is expected. The assessment of convergent validity is an iterative procedure; the more hypotheses tested, the more convincing the evidence that the instrument is valid [33]. In the absence of a gold standard, the convergent validity of the SarQol and its dimensions were assessed against other existing validated preference-based instruments (SarQol Overall and EQ Index; D1 Physical and mental health with EQVAS; D2 Locomotion with SPPB; D3 Body composition and MNA; D4 Functionality and Sarc-F; D5 ADL and Barthel and D6 Leisure Activities and IPAQ-METS). A significant but low to medium correlation (correlation range > 0.30–0.70) is indicative of good convergent validity, as related constructs are expected to be more strongly correlated than unrelated constructs. We decided against specifying an a priori hypothesis percentage criterion (such as 75%), as proposed by some authors to define the measurement tool as legitimate or responsive [34, 35], given the lack of specific instruments for the population with sarcopenia and the need to compare it with instruments validated in the general population. Indeed, only the Sarc-F scale is validated for the population with sarcopenia. As stated by the COSMIN authors themselves, “there is no criterion to decide whether an instrument is valid or responsive. Assessing validity or responsiveness is a continuous process of accumulating evidence” [33]. Convergent validity determines the relationship between the scale and other measures assessing the same construct. Some of the domains assessed with the SarQol were also evaluated using other tools: functionality was assessed using the Barthel scale, nutritional status using the MNA scale, and physical activity using the IPAQ. We evaluated the convergence by looking at the correlations between SarQol dimensions and assessment scales as well as between SarQol Overall and the EQ Index. The measure used was the correlation coefficient (Pearson’s or Spearman’s), which was assessed according to Feeny et al.’s criteria: high correlation, *r ≥ *0.50; moderate, r 0.35 to 0.49; and weak, *r ≤ *0.34 [36]. Divergent validity refers to the association between the scale and other measures that assess different constructs. We evaluated the divergence by looking at the correlation coefficient between SarQol and BMI, which hypothetically should be low (*r ≤ *0.30) [37].

### Statistical analysis

A second, independent researcher checked all data entered into the database. The normality of the quantitative variables was analyzed using the Kolmogorov–Smirnov test. Descriptive statistics were presented in the form of mean and standard deviation for normally distributed continuous variables and relative frequencies for categorical (qualitative) variables. The Kolmogorov–Smirnov test was used to assess normality. The ANOVA test for independent samples was used to compare means. Paired comparisons were performed using the Bonferroni test when the homogeneity requirement was met, and the Games–Howell test when it was not. The chi-squared test was used to compare categorical variables. The Youden Index (better values closer to 1) and area under the curve (AUC) were used to determine the cutoff value, where a value of 0.50 represents the level of randomness, and 0.90 is considered “excellent,” 0.80 “good,” 0.70 “fair,” and 0.60 “poor” [38]. We also calculated sensitivity, specificity, and the positive and negative predictive value of SarQol for sarcopenia and sarcopenic obesity. Statistical significance was defined as *p < *0.05. Statistical analysis was carried out with the SPSS Version 26.0 for Windows (IBM Corp., Armonk, NY, USA) and Jamovi 2.2.5 statistical packages.

## Results

After assessing sarcopenia and sarcopenic obesity in the 95 participants, 7.3% (*n = *7) were classified as having SO, 22.1% (*n = *21) as having sarcopenia, and 70.5% (*n = *67) as not having sarcopenia (Table 1).

**Table 1:** Characteristics of the sample

	Women without sarcopenia (*N = *67) Mean (SD)	Women with sarcopenia (*N = *21) Mean (SD)	Women with sarcopenic obesity (*N = *7) Mean (SD)	Total (*N = *95) Mean (SD)	*p*
Age, years	75.3 (5.2)	77.8 (6.6)	76.9 (6.0)	76.0 (5.7)	0.19
BMI, kg/m^2^	30.27 (5.83)	26.46 (3.54)	30.55 (3.79)	29.45 (5.48)	0.016^a^
Barthel, points	96.12 (7.17)	90.95 (9.01)	87.14 9.512	94.32 (11.29)	0.039^a^
MNA, points	26.64 (2.09)	24.69 (4.14)	24.21 (2.87)	26.03 (2.86)	0.004^a^
IPAQ, METS	2094.1 (2265.2)	1323.5 (1587.3)	1138.1 (908.4)	1834.8 (2065.1)	0.22
Short Physical Performance Battery, points	9.78 (2.24)	7.71 (2.76)	5.57 (1.9)	9.01 (2.65)	< 0.001^a,b^
Sarc-F, points	1.63 (1.73)	3.24 (2.36)	5.29 (0.95)	2.27 (2.13)	< 0.001^a,b^
Health-related quality of life, points					
EQ-5D-3L Index	0.73 (0.19)	0.54 (0.23)	0.54 (0.22)	0.67 (0.22)	< 0.001^a^
EQ-5D-3L VAS	69.03 (18.43)	62.14 (21.07)	57.14 (13.8)	66.63 (18.98)	0.14
SarQoL D1 Physical and Mental Health	70.55 (18.62)	66.21 (19.25)	58.37 (14.94)	68.28 (18.61)	0.24
SarQoL D2 Locomotion	67.02 (23.4)	54.63 (23.97)	34.92 (8.46)	60.81(24.48)	0.002^b^
SarQoL D3 Body Composition	68.33 (18.26)	73.84 (19.94)	61.67 (16.84)	69.06 (18.62)	0.31
SarQoL D4 Functionality	69.47 (18.9)	59.73 (19.63)	51.57 (5.94)	65.29 (19.09)	0.022^b^
SarQoL D5 ADL	61.56 (16.84)	45.78 (14.70)	31.19 (13.13)	54.66 (18.86)	< 0.001^a,b^
SarQoL D6 Leisure Activities	42.44 (19.55)	36.02 (14.25)	35.63 (6.29)	40.18 (17.59)	0.33
SarQoL D7 Fears	88.29 (13.64)	83.33 (13.56)	85.71 (16.81)	86.81 (13.9)	0.43
SarQoL Overall	66.51 (16.17)	56.59 (15.55)	45.09 (7.9)	61.95 (16.78)	0.001^b^
Comorbidities	*N* (%)	*N* (%)	*N* (%)	*N* (%)	
Respiratory	4 (5.97)	4 (19.05)	0 (0)	8 (8.4)	0.120
Cardiovascular	28 (41.8)	11 (52.38)	3 (42.8)	42 (44.21)	0.608
Metabolic	7 (10.44)	6 (28.57)	2 (28.6)	15 (15.79)	0.077
Functional disabilities (Barthel < 60 points)	1 (1.5)	1 (4.76)	0 (0)	2 (2.1)	0.609

*BMI* body mass index; *D* Dimension, *IPAQ* International Physical Activity Questionnaire; *MNA* mini nutritional assessment scale; *MET* metabolic equivalent of tasks; *VAS* visual analog scale

^a^Between women with sarcopenic and women without sarcopenia

^b^Between women with sarcopenic obesity and women without sarcopenia

Although the women with sarcopenia were slightly older, these differences were not statistically significant. There were significant differences in BMI, functionality (Barthel index), nutritional assessment (MNA), and Sarc-F.

Regarding quality of life, between-group differences were observed for the EQ-5D-3L index and the SarQol Overall, but not for the EQ-5D-3L VAS. Differences were also found in three of the SarQol dimensions: locomotion, functionality, and activities of daily living (Table 1).

The metric properties of the SarQol assessment instrument were analyzed, and acceptable results were obtained for all items assessed (Table 2). The percentages of data that were missing and computable were adequate, as were the values found after analyzing acceptability. Reliability and validity were also adequate in the sample analyzed. All the dimensions of the SarQol were significantly correlated with scales that also assess the same dimensions and with the overall score (Table 2).

**Table 2:** Metric properties of SarQol questionnaire in the sample

Property	Criteria	Results
Feasibility	Percentage of missing data (should be < 10%)	7.6%
	Percentage of computable data (should be > 95%)	98%
Acceptability	Mean, median, and standard deviation similar across items (15% maximum divergence)	Mean (SD) = 61.95 (16.78) Median* = *62.69
	Asymmetry and kurtosis should oscillate between − 1 and 1	Asymmetry (SE) = − 0.173 (0.283) Kurtosis (SE) = − 0.603 (0.559)
	Floor and ceiling effect (percentage of scores in the lower and upper extremes should be < 15%)	Floor effect = 0% Ceiling effect = 0%
Construct Validity	Convergence: Correlation between SarQol dimensions and assessment scales Convergence: Correlation between SarQol Overall and EQ Index	D1 Physical and mental health and EQVAS r = 0.490, *p < *0.001 D2 Locomotion and SPPB *ρ = *0.69, *p < *0.001 D3 Body composition and MNA *ρ = *0.36, *p = *0.002 D4 Functionality and Sarc-F *r = *− 0.76, *p < *0.001 D5 ADL and Barthel r = 0.46, *p < *0.001 D6 Leisure Activities and METS *ρ = *0.45, *p < *0.001 SarQol Overall and EQ Index *ρ = *0.62 *p < *0.001
	Divergence: correlation between SarQol and BMI	SarQol and BMI *r = *− 0.075, *p = *0.54
Reliability	Internal consistency: Cronbach’s alpha (≥ 0.7)	Cronbach’s alpha = 0.89

*ADL* activities of daily living; *BMI* body mass index; *D* Dimension; *ICC* interclass correlation index; *MET* metabolic equivalent of tasks; *MNA* mini nutritional assessment scale; *SE* standard error; *SPPB* short physical performance battery

**Fig. 2:**
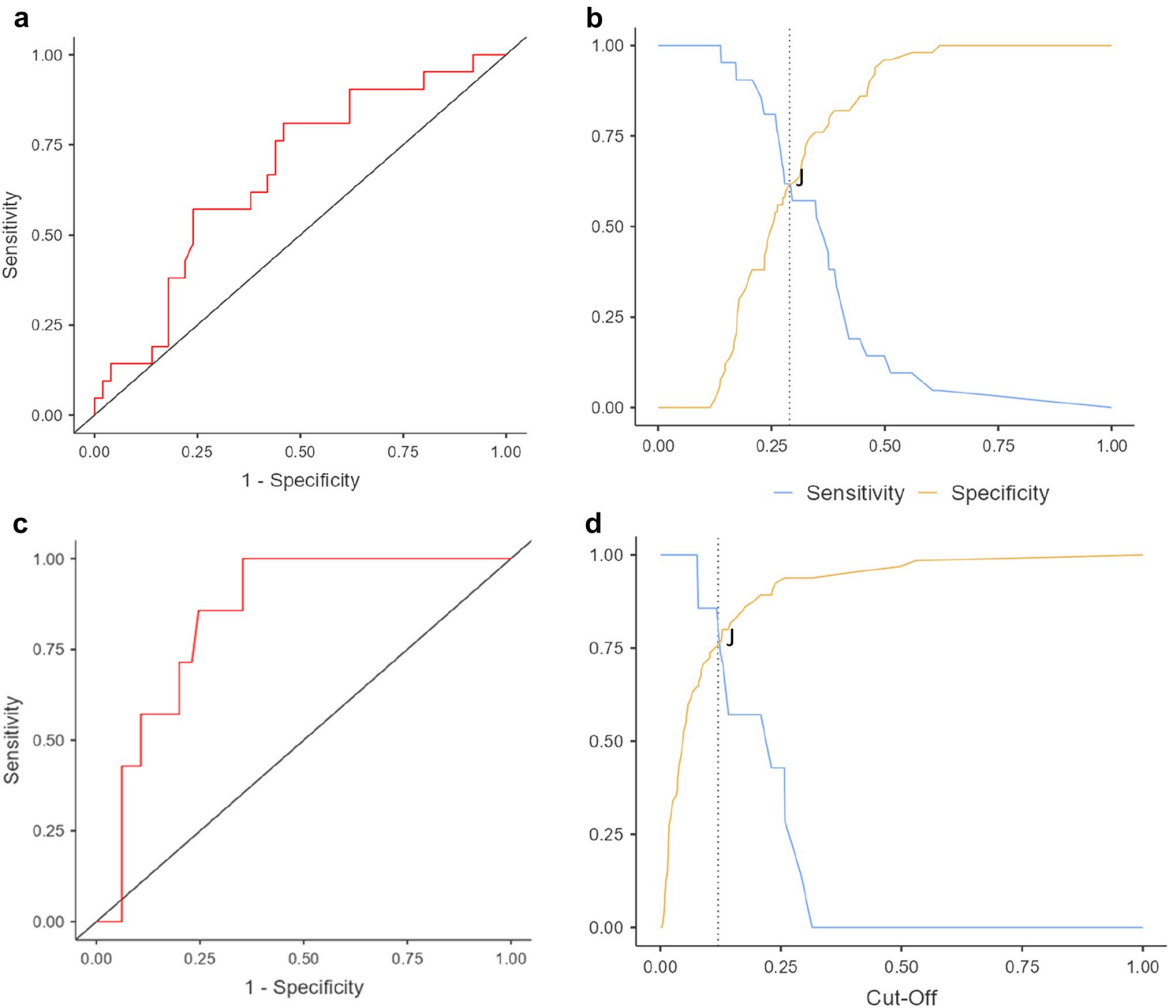
SarQol area under the curve and cutoff for sarcopenia and sarcopenic obesity in women aged 70 years or older. **a** SarQol area under the curve for sarcopenic women; **b** SarQol cutoff for sarcopenic women (Youden Index, J); **c** SarQol area under the curve for women with sarcopenic obesity; **d** SarQol cutoff for women with sarcopenic obesity (Youden Index, J)

After verifying that the SarQol scale had adequate metric properties, a cutoff was proposed for sarcopenia (≤ 60 points; *J = *0.999) and sarcopenic obesity (≤ 50 points; *J = *0.945) in women (Table 3 and Fig. 2).

**Table 3:** Diagnostic accuracy of SarQol questionnaire for sarcopenia and sarcopenic obesity cutoffs

Condition	SarQol Cutoff	AUC (95% CI)	Sensitivity % (95% CI)	Specificity % (95% CI)	PPV % (95% CI)	NPV % (95% CI)
Sarcopenia	≤ 60 points	0.67 (0.53–0.8)	61.9 (45–79)	62 (45–79)	40.62 (24–58)	79.49 (66–93)
Sarcopenic obesity	≤ 50 points	0.85 (0.74–0.95)	71.4 (52–91)	76.9 (58–96)	25 (6–44)	96.15 (87–99)

*AUC* Area under the curve; *CI* confidence interval; *NPV* negative predictive value; *PPV* positive predictive value

## Discussion

Sarcopenia and especially sarcopenic obesity are associated with lower health-related quality of life, as assessed by the SarQol. Using this tool, a cutoff of 60 points or less would be apt for screening women for sarcopenia, and a score of 50 points or less would allow screening women for sarcopenic obesity.

The prevalence of sarcopenia in our study (22.1%) is higher than that reported for older women in a recent meta-analysis, where the prevalence using the EWGSOP2 criteria was 2% [39]. This difference may be due to the fact that the prevalence estimate was obtained from just two studies of those included in the meta-analysis [39]. Furthermore, there may be possible variations by race, age, or study setting, as similar figures are observed in similar populations [18].

The prevalence of sarcopenic obesity in our sample was 7.3%. Given the recentness of the criteria used to diagnose SO in our sample [10], no other studies were available that have applied them to populations of a comparable size. However, studies estimating SO in older women using criteria predating those established by ESPEN/EASO have estimated that prevalence ranges from 2.8% to 19.64% in this population [28, 38–41]. This high variability is probably related to the use of differing and non-specific diagnostic criteria. Thus, we consider that our study, one of the first to apply the new ESPEN/EASO criteria, can contribute to systematizing diagnosis of SO and producing prevalence estimates that are comparable across different contexts and population groups.

Quality of life is an individual’s subjective perception of satisfaction or happiness with life in domains important to them, including but not limited to the health domain. So, HRQoL can be defined as “the subjective assessment of the impact of disease and treatment across the physical, psychological, social and somatic domains of functioning and well-being” [42].

An analysis of the metric properties of the SarQol instrument showed very similar values between the original version and the Spanish version [15, 19], confirming the appropriateness of the assessment instrument in our sample. Our results show adequate feasibility, acceptability, and reliability—very similar to the Spanish validation study [19]. Convergent validity showed significant correlations between the SarQol dimensions and the rating scales that converge with these dimensions. The assessment scales used for the convergent analysis confer an intrinsic validity to the results obtained, since in our study we observed a trend of greater effects among the groups, with the highest scores observed in the women without sarcopenia, followed by those with sarcopenia, and finally in those with SO. Significant between-group differences were found in the mean Barthel index, with the scores dipping below 90 points only in the SO group, showing worse functionality compared to the group with sarcopenia alone. This difference was also observed in the SarQol domain for activities of daily living, and there was a significant correlation between the SarQol and Barthel. Although this finding may sound obvious, previous studies measuring the Barthel index in different settings [43, 44] failed to show that SO, diagnosed using the previous set of criteria, worsened the ability to perform activities of daily living compared to the presence of sarcopenia alone. During aging, increased fat mass may exert a protective effect. Obesity has been said to have protective effects on disability, hospitalization, and risk of death in older adults, known as “the obesity paradox” [45]. Despite this, obesity and sarcopenia have traditionally been considered to be independently associated with adverse outcomes in older adults, but those who meet both criteria do not necessarily have an increased risk of disability [46]. Some studies report worse functionality in people with sarcopenic obesity [47, 48] and others in those with sarcopenia [49, 50]. In no previous study has SO been diagnosed using the ESPEN/EASO criteria, which makes it difficult to compare results. The diagnostic criteria used in this study (EWGSOP2 for sarcopenia and ESPEN/EASO for SO) could be considered superior for diagnosing sarcopenic obesity in our sample and therefore for better *characterizing* the condition. The superiority of these criteria in the diagnosis of SO could call into question the obesity paradox, raising doubts at least regarding obesity-related functionality and quality of life, but it is essential to carry out more studies in this direction.

In the nutritional assessment carried out with the MNA scale, as well as with the SarQol body composition dimension, the SO group obtained the worst scores, showing a tendency to have worse nutritional status relative to women with sarcopenia alone. These results, although falling short of statistical significance, could be relevant since they differ from those reported by other studies that diagnosed SO according to the older criteria, which reported worse nutritional status in people with sarcopenia compared to sarcopenic obesity [14, 51]. Our results may diverge because our sample was composed exclusively of women, but above all, because other studies have used the appendicular lean mass to BMI ratio [51] instead of the skeletal muscle mass to weight ratio, which is recommended by ESPEN/EASO as the weighting parameter for muscle mass when measured by BIA, and which we used in the present study. Adoption of these updated criteria in future research will enable a more reliable comparison of results.

The same, significant between-group differences were observed in the values obtained with SPPB and Sarc-F. The women with SO presented the lowest values in SPPB and the highest in Sarc-F, indicating more severe impacts. These differences were also observed in the dimensions of locomotion and functionality, which correlate with SPPB and Sarc-F, respectively. Previous studies in the community population corroborate this association [52–54], although other studies have also found worse physical performance in the population with sarcopenia [55]. The existence of this association is not entirely clear, but factors such as the mean age of the sample, their sex, and the sarcopenia or sarcopenic obesity endpoints could affect these results [56].

Assessing HRQoL with a specific instrument enables us to find differences between groups within the same disease group. Our results show that the women with SO have the worst HRQoL (45.09), followed by the women with sarcopenia (56.59) and finally those without sarcopenia (66.51). These values are lower than those found in similar populations in terms of sex, age, and geographical area [18]. To our knowledge, there are no published studies analyzing QoL in SO using the ESPEN/EASO criteria, although there are studies reporting that the loss of QoL in SO assessed with other criteria is high [11, 57, 58]. Comorbidities often associated with SO, such as osteoporosis or cardiovascular risk associated with obesity, together with changes in the aging process itself, could have an effect in this regard [59]. Previous studies indicate that SO is even associated with alterations in the social environment, given that pain, fear of falls, and fractures all limit activity, which leads to a loss of quality of life [60, 61].

Our findings evidence the loss of QoL in the group of women with SO, who obtained the lowest scores overall and in all dimensions except Fears. Although previous studies found no differences between sarcopenia and sarcopenic obesity [13], or even the opposite direction of effect, in favor of SO [14], the previous lack of consensus in the definition of SO could have represented an important limitation. Our results confirm that the new diagnostic pathway proposed by ESPEN/EASO [10] would correctly identify women with SO.

The differences that the SarQol tool revealed between sarcopenia and SO are not evident with a generic instrument, such as the EQ5D index, where the HRQoL in women with both sarcopenia and SO shows similar values (0.54), though lower than those in the non-sarcopenic group (0.73). Generic quality of life assessment instruments are useful for large populations or where the aim is to evaluate an intervention for a general population. But in the case of a population with a specific disease or condition, they may not be as sensitive, as this condition would bias the results [62].

The specific SarQol tool is useful for assessing HRQoL in people with sarcopenia, whether cross-sectionally or longitudinally [63]. The results obtained in different studies with similar metric properties corroborate and strengthen the validity of the results found in different populations [15]. Future studies can thus assess the success of interventions in the sarcopenic population both in terms of the reversibility of the criteria and in terms of QoL.

Guillamón-Escudero et al. [18] found the same cutoff point as we did, of 60 points or below, for sarcopenia screening in community-dwelling older people, with a very similar AUC. Furthermore, our results provide similar sensitivity and specificity data to those published in the French-speaking population [17]. Regarding SO, ours is the first study to propose a cutoff of 50 points or less on the SarQol, with an AUC higher than that of sarcopenia alone and with better diagnostic accuracy values. It is also the first study to analyze the prevalence of sarcopenic obesity in a population of women using the ESPEN/EASO criteria.

Early identification of people with sarcopenia and sarcopenic obesity is of vital importance in the clinical setting, as it enables prompt interventions for preventing and reversing the course. But the clinical reality is that a comprehensive assessment of all the criteria for sarcopenia and sarcopenic obesity is time and resource intensive. Screening tools based on self-report are far more cost effective. Our results suggest that SarQol could be another screening tool that enables quick identification of people at risk of sarcopenia and sarcopenic obesity, as well as providing QoL data that is so important for assessing the effects of health interventions in general. The concordance of our results with other studies on sarcopenia validates the use of the tool for screening [17–19] and also reinforces the results obtained on sarcopenic obesity.

This study has several limitations, the first being that only women were assessed, so the cutoff may not be generalizable to male populations. Likewise, our population was community based, so the values may not be appropriate for analyzing institutionalized people. Due to the small number of women who have sarcopenic obesity, investigations with a bigger sample size would be required to support the findings. The cutoffs were calculated based on the EWGSOP2 and ESPEN/EASO sarcopenia criteria for SO, so an assessment with other criteria could also produce variations in these values. Further studies analyzing the metric properties of this instrument are needed to reinforce the results obtained in the present study.

## Conclusions

Sarcopenia, and especially sarcopenic obesity, leads to a loss of quality of life in women. The SarQol assessment instrument could help in screening for sarcopenia following the EWGSOP2 criteria (scores of ≤ 60 points) and for sarcopenic obesity following the ESPEN/EASO criteria (scores of ≤ 50 points).

## Data Availability

The datasets generated during and/or analyzed during the current study are available from the corresponding author on reasonable request.
